# Individual Differences in Cognitive Function in Older Adults Predicted by Neuronal Selectivity at Corresponding Brain Regions

**DOI:** 10.3389/fnagi.2017.00103

**Published:** 2017-04-18

**Authors:** Xiong Jiang, Jessica R. Petok, Darlene V. Howard, James H. Howard

**Affiliations:** ^1^Department of Neuroscience, Georgetown UniversityWashington, DC, USA; ^2^Department of Psychology, Georgetown UniversityWashington, DC, USA; ^3^Department of Psychology, St. Olaf CollegeNorthfield, MN, USA; ^4^Center for Brain Plasticity and Recovery, Georgetown University Medical CenterWashington, DC, USA; ^5^Department of Psychology, Catholic University of AmericaWashington, DC, USA

**Keywords:** neuronal selectivity, episodic memory, verbal fluency, hcorr, aging

## Abstract

Relating individual differences in cognitive abilities to neural substrates in older adults is of significant scientific and clinical interest, but remains a major challenge. Previous functional magnetic resonance imaging (fMRI) studies of cognitive aging have mainly focused on the amplitude of fMRI response, which does not measure neuronal selectivity and has led to some conflicting findings. Here, using local regional heterogeneity analysis, or *H*_*corr*_, a novel fMRI analysis technique developed to probe the sparseness of neuronal activations as an indirect measure of neuronal selectivity, we found that individual differences in two different cognitive functions, episodic memory and letter verbal fluency, are selectively related to *H*_*corr*_-estimated neuronal selectivity at their corresponding brain regions (hippocampus and visual-word form area, respectively). This suggests a direct relationship between cognitive function and neuronal selectivity at the corresponding brain regions in healthy older adults, which in turn suggests that age-related neural dedifferentiation might *contribute to* rather than *compensate for* cognitive decline in healthy older adults. Additionally, the capability to estimate neuronal selectivity across brain regions with a single data set and link them to cognitive performance suggests that, compared to fMRI-adaptation—the established fMRI technique to assess neuronal selectivity, *H*_*corr*_ might be a better alternative in studying normal aging and neurodegenerative diseases, both of which are associated with widespread changes across the brain.

## Introduction

Cognitive abilities are highly heterogeneous among individuals, and this variance is typically even higher in older compared to middle-aged adults (Morse, 1993; Spreng et al., 2010). Because individuals with lower cognitive performance are at a higher risk of Alzheimer's disease (AD) and other age-related dementias (Masur et al., 1994; Albert et al., 2001; Riley et al., 2005), identifying the neural bases of these individual cognitive differences in older adults might reveal potential neural targets for interventional therapies. These could in turn help to preserve or even improve cognitive function, thereby reducing the risk of AD and other types of dementia. However, relating individual differences in cognitive performance to neural substrate remains a major challenge.

Previous work on object recognition has suggested that individual differences in behavioral performance might be accounted for by differences in neuronal selectivity in corresponding brain regions, with higher neuronal selectivity associated with higher behavioral performance, and vice versa (Sigala et al., 2002; Freedman et al., 2006; Jiang et al., 2007). Meanwhile, computational simulation and theoretical works have suggested that aging leads to a decrease in neuronal selectivity, often referred to as neural “dedifferentiation” (Li et al., 2001; Park and Reuter-Lorenz, 2009). This neural dedifferentiation in aging is thought to be a key contributor to cognitive decline in older adults (Li et al., 2001). That is, neurons in the aged-brain become less selective, leading to increasing overlap in the neuronal response to different stimuli. This, in turn, contributes to diminished behavioral performance as well as to an increase in the workload of executive/attention circuits. Recent single-unit recording studies in animals (Schmolesky et al., 2000) and functional magnetic resonance imaging (fMRI) studies in human subjects (Goh et al., 2010; Lee et al., 2011; Park et al., 2012) have lent support to this neural dedifferentiation theory of aging. For instance, using fMRI-adaptation (fMRI-A) and morphed face stimuli with varying shape similarity, two independent groups of researchers showed that, compared to young adults, neuronal selectivity in the fusiform face area, or FFA, a critical region in face processing (Kanwisher et al., 1997), is reduced in healthy older adults (Goh et al., 2010; Lee et al., 2011). In addition, using a different fMRI technique, multi-voxel pattern analysis (MVPA) (Norman et al., 2006), it has been shown that the distinctiveness of neuronal response to preferred vs. non-preferred stimulus classes is reduced in aged brain (Carp et al., 2010). Taken together, these studies reveal a general reduction in the distinctiveness of neural representations with healthy aging, i.e., age-related neural dedifferentiation.

While these previous studies have provided converging evidence in support of the age-related neural dedifferentiation theory, one key element is missing. To our knowledge, none of these studies has established a direct relationship between cognitive function and neuronal selectivity (or neural distinctiveness Carp et al., 2010) at corresponding brain regions in older adults. In other words, it is not clear whether the individual differences in neuronal selectivity in older adults can be used to predict individual differences in cognition, thereby providing some insight into inter-individual heterogeneity of cognitive function with age.

Recent theoretical studies of aging—inspired by findings of increased activity and/or recruitment of additional brain regions in functional neuroimaging studies of healthy older adults—propose that neural dedifferentiation might serve as a compensatory mechanism in older adults. That is, neurons in the aged brain may become more broadly tuned so that they might be recruited to support cognitive process in other tasks (Park and Reuter-Lorenz, 2009; Reuter-Lorenz and Park, 2010; but see Reuter-Lorenz and Park, 2014), suggesting the decrease in neuronal selectivity in aging brain might be beneficial, that is, it might help to *compensate for* cognitive decline in older adults.

In direct contrast to this idea that dedifferentiation *compensates for cognitive decline*, studies of object recognition suggest that lower neuronal selectivity is associated with lower cognitive performance, i.e., a positive correlation between neuronal selectivity and cognitive performance (Freedman et al., 2006; Jiang et al., 2007, 2013; Scholl et al., 2014). That is, the decrease in neuronal selectivity in aging brain *contributes to* rather than *compensate for* cognitive decline in older adults. Furthermore, experimental data and computational models of object recognition suggest that a decrease in neuronal selectivity would lead to an increase in unspecific neuronal response (Freedman et al., 2006; Jiang et al., 2006), suggesting that the increase in fMRI signal in additional brain regions often seen in older adults might be—at least partially—due to the decrease in neuronal selectivity (in addition to modulations due to attention, task difficulty, and effort), as *a consequence of cognitive decline rather than a compensation to cognitive decline*. Thus, additional research is warranted to investigate the relationship between age-related neural dedifferentiation, general age-related cognitive decline, and individual differences, and to reconcile the inconsistency between the two competing hypotheses.

Here we studied this question by examining the relationship between different cognitive functions and neuronal selectivity at corresponding brain regions in older adults, using a novel fMRI data analysis technique, *local regional heterogeneity analysis*, or *H*_*corr*_, which we recently developed to estimate neuronal selectivity based on fMRI activation patterns (Jiang et al., 2013). Briefly, this technique calculates the variance (heterogeneity) of local voxel-wise correlations in a region of interest (ROI), also termed *H*_*corr*_, to assess the sparseness of neuronal activations as an indirect measure of neuronal selectivity. Higher *H*_*corr*_ indicates larger inter-voxel variance and hence greater neuronal sparseness, which in turn suggests sharper neuronal tuning, and vice versa. This technique is motivated by earlier computational, behavioral, imaging, and single-unit studies that suggest the sparseness of activation patterns is related to neuron selectivity (Freedman et al., 2006; Jiang et al., 2006): neurons with high selectivity produce a sparse neural code, as each neuron only responds to a small set of stimuli that are highly similar to its preferred stimulus. In contrast, less selective neurons respond to a greater number of dissimilar stimuli, leading to greater overlap in responses and less sparse neural representations. For instance, single-unit studies in monkeys have shown that learning produces sparser codes, with neurons responding to fewer stimuli after training (Kobatake et al., 1998; Freedman et al., 2006). Therefore, when neuronal selectivity is measured using our technique with fMRI, a lower local regional heterogeneity of correlations (*H*_*corr*_) should be associated with a lower neuronal selectivity and thus a poorer behavioral performance. Conversely, greater heterogeneity implies higher neuronal selectivity and thus better behavioral performance across subjects. Indeed, we have found that behavioral face discrimination in adults with autism spectrum disorders can be quantitatively predicted by neuronal selectivity in FFA, estimated via both the novel *H*_*corr*_ and the established fMRI-A techniques (Jiang et al., 2013). This suggests that *H*_*corr*_ reliably estimates neuronal selectivity.

In the present study, we investigated whether the novel *H*_*corr*_ technique can effectively assess neuronal selectivity across brain regions in older adults and whether the *H*_*corr*_-estimated neuronal selectivity can selectively predict individual differences in behavioral performance on two cognitive functions, episodic memory and letter verbal fluency. Both of these show declines in healthy aging (Fleischman et al., 2004; Cansino, 2009; Elgamal et al., 2011; Stokholm et al., 2013) as well as in neurodegenerative disease, such as AD (Henry et al., 2004; Rémy et al., 2005). Specifically, we used *H*_*corr*_ to assess neuronal selectivity at two brain regions, the hippocampus, which is associated with episodic memory (Tulving, 2002; Squire and Wixted, 2011), and the visual word form area (VWFA), an area in the left ventral occipitotemporal cortex that is important for lexical aspects of language skills (McCandliss et al., 2003) and has been associated with letter verbal fluency (Gleissner and Elger, 2001). Critically, based on previous studies of object recognition in animal and human subjects and computational modeling, we predicted a double dissociation, such that *H*_*corr*_ at the hippocampus would predict individual differences in episodic memory, but not letter verbal fluency, whereas *H*_*corr*_ at the VWFA would show the opposite pattern. Further, we predict a positive correlation in both cases. In addition, it would predict there is no correlation between letter verbal fluency performance and *H*_*corr*_ at the VWFA homologous region in the right hemisphere (R-VWFA), which is typically associated with face processing in young adults (Kanwisher et al., 1997). By contrast, the neural dedifferentiation-related compensation theory would predict a negative correlation between cognitive performance and *H*_*corr*_ at other brain regions, i.e., a better performance in letter verbal fluency would correlate with a lower *H*_*corr*_ at R-VWFA. That is, while R-VWFA and nearby regions are typically associated with face processing, the reduced neuronal selectivity due to age-related neural dedifferentiation might help to recruit neurons in this region to assist language processing (e.g., letter verbal fluency) in healthy older adults, with a direct contrast to the prediction of the computational theories of object recognition. Here we tested the two hypotheses by applying the novel *H*_*corr*_ technique to a previously collected data set (Simon et al., 2011).

## Experimental procedures

### Participants

Twelve healthy older adults (age range: 63–72 years old, mean age: 67.5 ± 3.2 years old, nine women) participated in the study. Experimental procedures were approved by Georgetown University's Institutional Review Board, and written informed consent was obtained from all subjects prior to the experiment. The data from one additional subject were excluded due to missing data.

Participants were screened for MRI safety, neurological disease or disorder, and drugs known to influence cognition. In addition, subjects were excluded from the study if they met criteria for dementia (i.e., a score of below 27 on the Mini-Mental State Examination) or had abnormal intelligence status (i.e., scores outside the expected age range on neuropsychological measures of processing speed, cued recall, free recall, verbal memory, vocabulary, and reading ability (n = 0).

The demographic info and neuropsychological test scores are shown in Table 1.

**Table 1:** **Demographic and neuropsychological data (n = 12)**.

**Demographic**	**Range**	**Mean (STD)**
Age	63, 72	67.5 (3.2)
Gender	9 Female, 3 Male	
Education	12, 21	17.2 (2.4)
**Neuropsychological tests**	**Scores**	
MMSE	28, 30	29.3 (0.8)
^*^COWAT-FAS Sum	27, 65	46.0 (12.5)
WAIS-III vocabulary	57, 77	67.9 (5.7)
WAIS-III digit symbol coding	31, 83	60.7 (13.7)
WAIS-III digit symbol pairing	4, 18	10.4 (5.0)
WAIS-III digit symbol recall	5, 9	7.0 (1.4)
^*^WAIS-III logical memory (unit)	34, 55	45.8 (6.4)
WAIS-III logical memory (thematic)	16, 21	19.0 (2.0)
WAIS-III digit span forward	9, 15	11.8 (2.0)
WAIS-III digit span backward	4, 12	9.0 (2.6)
WJ-III Word Attack SS	24, 29	27.5 (1.4)
^*^WJ-III Word Identification SS	70, 76	74.2 (1.7)
USC-REMT free recall correct	15, 37	24.9 (5.1)
USC-REMT free recall repetitions	90, 13	2.7 (3.6)
USC-REMT free recall intrusions	0, 2	1.0 (0.9)

*MMSE, Mini-Mental State Examination; WAIS-III, Wechsler Adult Intelligence Scale, 3rd ed.; COWAT-FAS, Controlled Oral Word Association Test-FAS; USC-REMT, University of Southern California-Repeatable Episodic Memory Test; WJ-III, Woodcock-Johnson, 3rd ed*.

* *Tests used for the H_corr_ correlation analyses*.

### Neuropsychological tests

A comprehensive neuropsychological test battery was administered to all participants 1 day or 2 days after the MRI scan (see Table 1 for the complete list). Here we focused on episodic memory performance measured with the Logical Memory subtest of the Weschsler Memory Scale–Third Edition (WMS-III), and language skills measured with FAS verbal fluency and Wood-Johnson III Word Identification test.

### Implicit sequence learning scans

**Figure 1:**
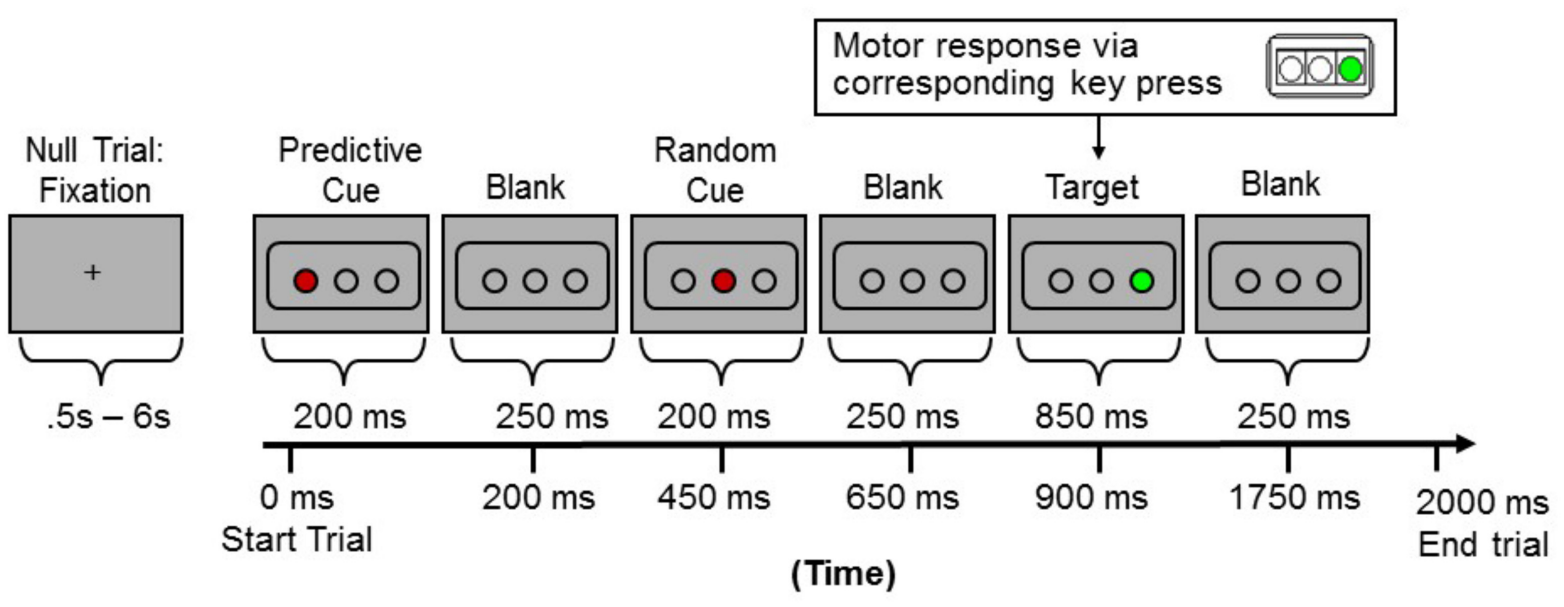
**Sample presentation of one trial (or “triplet”) during the event-related fMRI scans**.

The Hcorr data were derived from MRI images collected during an event-related design consisting of three runs for each subject while they performed a shortened and simplified version of the Triplets Learning Task (TLT) (Howard et al., 2008). The details of task design can be found elsewhere (Simon et al., 2011) and are not described here because the task itself is not the focus of the current paper. Briefly, three open circles were displayed against a gray background on the screen (Figure 1). Each trial or “triplet” began with two sequentially presented cue events (circles filling in red—displayed for 200 ms each), which were followed by a target (a circle filling in green—displayed for 850 ms). Each event was immediately followed by a 250 ms blank screen, and each trial lasted 2000 ms. Participants passively viewed the first two red events and responded to the third, green target event location as quickly and as accurately as possible via a corresponding button (one of three buttons on a button box held in the right hand). Each run consisted of 135 trials and lasted 6 m and 30 s. Within each run, trial orders and trial durations were implemented using OptSeq2 (Dale, 1999), resulting in a rapid event-related design with a temporally jittered intertrial interval (0.5–6 s, mean = 1.36 s). The overall accuracy was 98.4 ± 0.04%, and the reaction time was 482.72 ± 41.76 ms.

### MRI data acquisition and analysis

MRI data were acquired at Georgetown University's Center for Functional and Molecular Imaging using an echo-planar imaging (EPI) sequence on a 3.0 Tesla Siemens Trio scanner (Flip angle = 90°, *TR* = 2.5 s, *TE* = 30 ms, FOV = 256 × 256 mm, 64 × 64 matrix) with a twelve-channel head coil. Fifty descending axial slices (thickness = 3.7 mm, 0.3 mm gap; in-plane resolution = 4.0 × 4.0 mm^2^) were acquired. At the end, three-dimensional T1-weighted MPRAGE images (resolution 1 × 1 × 1 mm^3^) were acquired from each subject.

The EPI images were spatially realigned and unwrapped using the SPM2 software package (http://www.fil.ion.ucl.ac.uk/spm/software/spm2/), then all images were resliced to 2 × 2 × 2 mm^3^, normalized to a standard MNI reference brain in Talairach space, and smoothed with 6 mm Gaussian kernel using SPM2.

The hippocampal formation and parahippocampal regions of interest (ROIs) were defined using the AAL toolbox (Tzourio-Mazoyer et al., 2002), and the voxels from the surface of ROIs were removed before analyzing data to limit the possibilities of including voxels from other nearby regions. The VWFA ROI was defined as a 4-mm sphere centered at (MNI: −42 −54 −18) (Glezer et al., 2009), and right-hemispheric symmetrical region (R-VWFA) was defined as a 4-mm sphere centered at (MNI: 42 −54 −18), though similar results were obtained when the VWFA and R-VWFA ROIs were defined with different radius (3 or 5 mm).

Results using conventional fMRI data analysis can be found elsewhere (Simon et al., 2011). Here we reported results using a novel fMRI data analysis technique (see below).

### Local regional heterogeneity analysis

For the local regional heterogeneity and synchronization analysis, we first extracted the raw time series data in each of ROIs from the three runs of ER scans for each subject, followed by removing any linear trends and low frequency variations. The fMRI data were used in a pair-wise correlation analysis between each voxel, which resulted in a set of pairwise correlation coefficients (for *n* voxels), *r*_*ij*_.

(1) $$r_{ij}=corr(Vox_{i},Vox_{j}),\;i,j\in 1..n$$

We then calculated a measure of local heterogeneity, *H*_*corr*_, as the standard error of the mean (SEM) of those averaged correlation coefficients (*r*_*ij*_, *i* < *j*, because *r*_*ij*_ = *r*_*ji*_, and *r*_*ii*_ = 1).

(2) $$H_{corr}=\sqrt{\frac{\sum_{i=1}^{n-1}\sum_{j=i+1}^{n}{\left(r_{ij}-u\right)}^{2}}{N\times \left(N-1\right)}}\;where\;N\text{​}=\text{​}\sum_{i=1}^{n-1}i\;u=\frac{1}{N}\sum_{i=1}^{n-1}\sum_{j=i+1}^{n}r_{ij}$$

The *H*_*corr*_ of each individual run and each individual ROI was calculated separately, and the averaged values of three runs (and for hippocampus and parahippocampus, mean of both hemispheres) were used in the main analysis, but similar results were observed with data from each run and each hemisphere (see Supplementary Materials).

## Results

Three runs of fMRI data were acquired while subjects (N = 12) performed an implicit sequence learning task (Simon et al., 2011) (also see Figure 1). The neuronal selectivity at VWFA and bilateral hippocampal formation were estimated using the *H*_*corr*_ technique, and then correlated with verbal fluency and episodic memory. For comparison, *H*_*corr*_ at the bilateral parahippocampal region and the right-hemispheric symmetrical region of VWFA (R-VWFA) (Cohen et al., 2003) were also obtained (to serve as control regions) (see Experimental Procedures).

### Episodic memory performance is predicted by *H*_*corr*_ at hippocampus but not the VWFA and parahippocampal region

Episodic memory is one of the most commonly affected cognitive functions in elderly adults (Fleischman et al., 2004; Cansino, 2009). While it is well-known that reduced episodic memory is mainly driven by neuronal dysfunction in the hippocampal region, the exact neural mechanisms remain unknown. Given recent findings suggesting that neurons in hippocampus are highly selective with a sparse neural representation (Quiroga et al., 2005; Viskontas et al., 2009), we hypothesized that, in healthy older adults, poor episodic memory is due to reduced neuronal selectivity in the hippocampus and individual differences in episodic memory can be related to variations in neuronal selectivity in the hippocampus.

**Figure 2:**
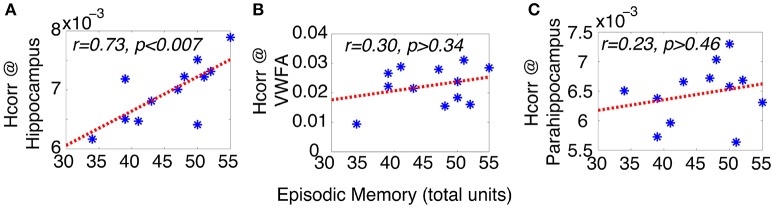
**Episodic memory performance of older adults can be predicted by *H*_*corr*_ at hippocampal formation (A)**, but not VWFA **(B)**, nor parahippocampal region **(C)**, suggesting the episodic memory performance in older adults is closely coupled with neuronal selectivity in hippocampus. ^*^Represents data from each individual subject.

To test this hypothesis, we examined the relationship between neuronal selectivity in hippocampus and episodic memory in these older adults. Episodic memory was assessed outside of the MRI scanner with the Logical Memory subtest (LMS) of the Weschsler Memory Scale–Third Edition (WMS-III), using the total unit score of immediate recall. Neuronal selectivity at the hippocampal region was estimated via *H*_*corr*_ (using the mean of both hemispheres and three runs, similar results were observed using data from each hemisphere and each run separately). Pearson's correlation analyses revealed that episodic memory performance was significantly correlated with *H*_*corr*_ in the hippocampus; with higher selectivity (i.e., a higher *H*_*corr*_ value) associated with better performance on episodic recall (total units, r = 0.73, p < 0.007, Figure 2A). In contrast, there was no significant correlation between episodic memory performance and the *H*_*corr*_ at either the VWFA (r = 0.30, p > 0.34, Figure 2B) or the parahippocampal region (r = 0.23, p > 0.46, Figure 2C). Thus, episodic memory performance in older adults is closely, and selectively, related to the neuronal selectivity in hippocampus as estimated by *H*_*corr*_.

The neural mechanisms underlying reduced episodic memory have been the focus of many neuroimaging studies of aging (Corkin, 1998; Chhatwal and Sperling, 2012), which generally suggest that neuronal dysfunction at hippocampus might underlie reduced episodic memory in older adults. For instance, reduced hippocampal volume has been linked to lower memory performance in both older adults and patients with probable AD (Petersen et al., 2000). However, the link between episodic memory deficits and neuronal dysfunction at hippocampus remains elusive. For instance, fMRI studies of episodic memory in healthy older adults have revealed conflicting reports; while some studies revealed decreased neural activity in hippocampus of older than younger adults (Daselaar et al., 2006; Antonova et al., 2009), others have reported the opposite (Yassa et al., 2011). These conflicting reports might reflect the technical limitations of conventional fMRI techniques: in fMRI, higher response amplitude can result from either fully functioning neurons or broadly tuned neurons (which will increase the number of neurons responding), while lower response amplitude may reflect loss of neurons responding or sharply tuned neurons (which will also lead to a decrease in number of neurons responding). These two scenarios have very different implications for performance, but cannot be differentiated with conventional fMRI techniques that rely on response amplitude, which does not measure neuronal selectivity. Furthermore, the relationship between individual differences in episodic memory and neuronal function in hippocampus remains an open question of high interest. In contrast, the *H*_*corr*_ technique we use here is sensitive to neuronal selectivity, and reveals a selective association between neuronal selectivity at hippocampus and a measure of episodic memory. This suggests that age-related decline in episodic memory might be related to region-specific age-related neural dedifferentiation, with a poorer episodic memory associated with a lower neuronal selectivity in hippocampus, and potentially a higher risk of dementia. These results are in line with a previous study of amnestic mild cognitive impairment (aMCI), which found that neuronal selectivity (estimated via fMRI-A) is lower in the hippocampus of aMCI than that of healthy controls (Johnson et al., 2004).

Moreover, here we found there was no significant correlation between episodic memory and *H*_*corr*_ at parahippocampal region. This finding is a bit surprising, as previous studies have suggested the involvement of parahippocampus in memory encoding and retrieval (Hayes et al., 2007). However, our lack of correlation in the present study is consistent with those showing that the parahippocampus is not related verbal or semantic related memory (what was assessed via LMS) but rather spatial memory processes (Moscovitch et al., 2006; Aminoff et al., 2013).

### Letter verbal fluency predicted by *H*_*corr*_ at VWFA but not hippocampus and R-VWFA

Verbal fluency tasks have been widely used to assess both language- and executive-related cognitive performance in several populations (McDowd et al., 2011), and studies have found that verbal fluency declines in healthy aging (Elgamal et al., 2011; Stokholm et al., 2013), as well as in Alzheimer's disease (Henry et al., 2004; Clark et al., 2009) and other neurological disorders (Henry and Beatty, 2006; McDowd et al., 2011). However, the neural bases of verbal fluency remain to be elucidated. For instance, while neuroimaging studies of letter and semantic verbal fluency have consistently revealed the involvement of left inferior frontal gyrus (Costafreda et al., 2006), supporting the critical role of executive function in verbal fluency tasks (Bolla et al., 1990), the majority of neuroimaging studies of letter and semantic verbal fluency have failed to reveal the involvement of the left ventral occipitotemporal region (including VWFA) (Costafreda et al., 2006; Wagner et al., 2014). This stands in contrast to findings from neuropsychological studies that have found lesions of the left but not right temporal lobe impair letter verbal fluency (Gleissner and Elger, 2001). Furthermore, using a different experimental design, a recent fMRI study (Birn et al., 2010) reported significant activations in VWFA (MNI: −45 −51 −11) related to letter verbal fluency tests, consistent with the lesion studies. Nevertheless, the functional role and relationship between verbal fluency and VWFA are still poorly understood. Given the previous findings suggesting the critical role of VWFA in language (McCandliss et al., 2003) and the sharp tuning of VWFA neurons (Glezer et al., 2009), here we hypothesized that there is a relationship between neuronal selectivity in the VWFA and verbal fluency, whereby poorer verbal fluency in older adults is related to reduced neuronal selectivity in VWFA.

**Figure 3:**
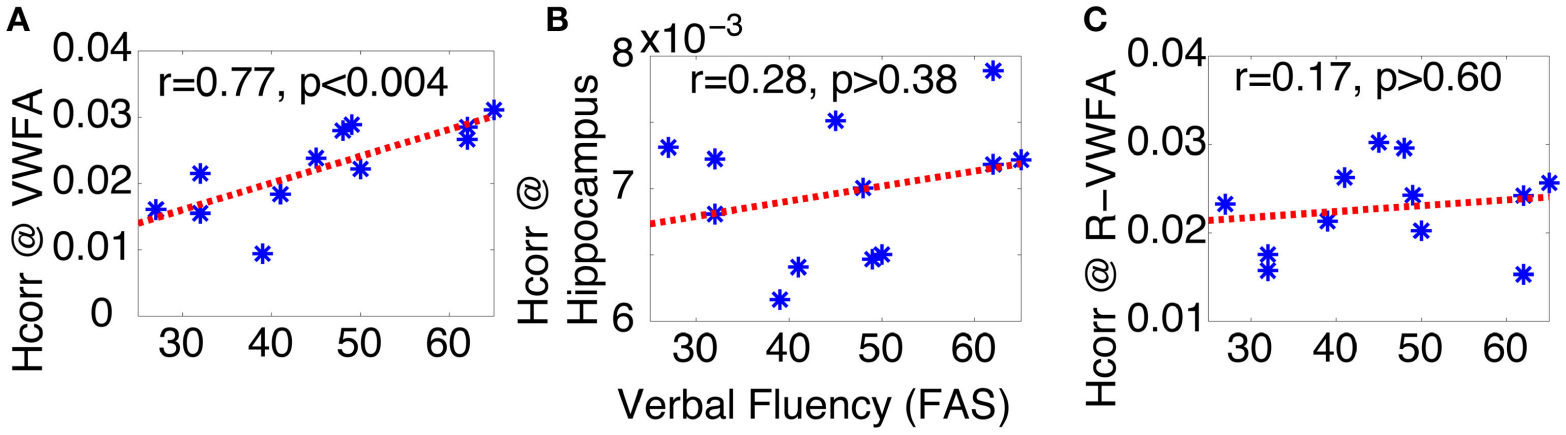
**Verbal fluency (FAS) of older adults can be predicted by *H*_*corr*_ in the VWFA (A)**, but not hippocampus **(B)**, nor the R-VWFA **(C)**, confirming the key role of VWFA in language skills. ^*^Represents data from each individual subject.

**Figure 4:**
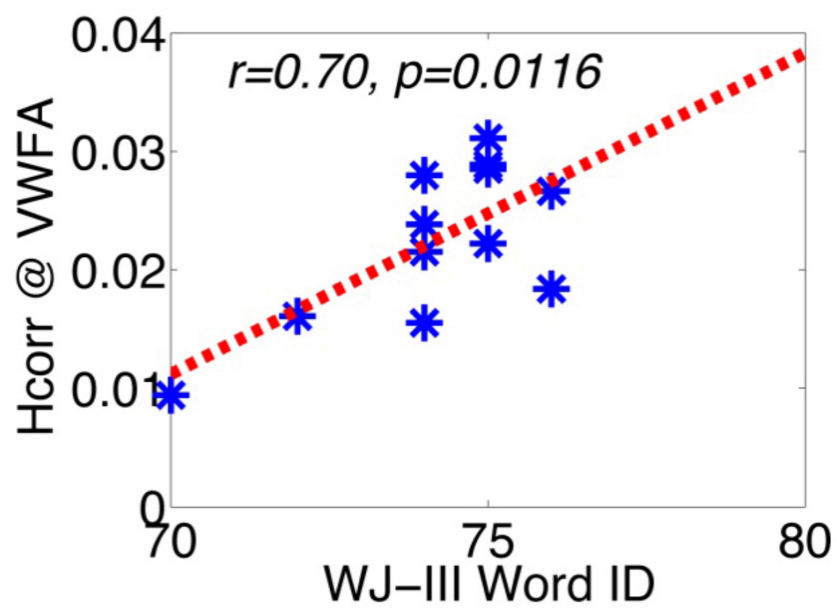
**The correlation between WJ-III Word ID and *H*_*corr*_ in VWFA**. ^*^Represents data from each individual subject.

**Figure 5:**
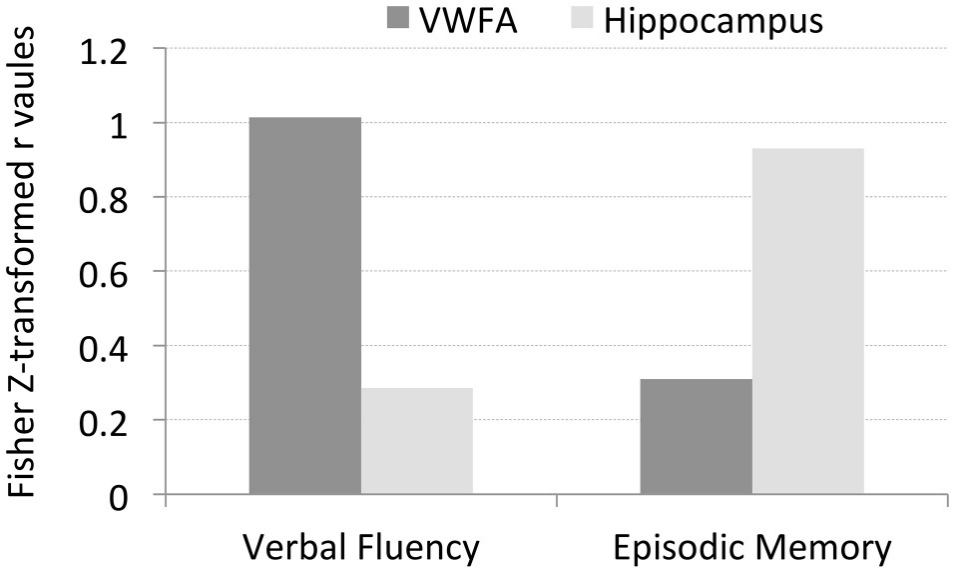
**The Z-transformed correlation coefficients between performance of two cognitive functions (episodic memory and verbal fluency) and *H*_*corr*_ at two brain regions (hippocampus and VWFA), respectively**. The data suggests a double disassociation, that is, *H*_*corr*_ at hippocampus predicts episodic memory but not verbal fluency, while *H*_*corr*_ at VWFA predicts verbal fluency but not episodic memory.

To test these hypotheses, we examined the relationship between letter verbal fluency and *H*_*corr*_ in VWFA and other comparison brain regions. Verbal fluency was assessed with the letter verbal fluency test (FAS), measured as the total number of words beginning with a given letter (F, A, and S) reported within 1 min. Pearson's correlation analyses revealed a significant correlation between verbal fluency and *H*_*corr*_ at VWFA (r = 0.77, p < 0.004, Figure 3A), but not at the hippocampus (r = 0.28, p > 0.38, Figure 3B). In addition, there was no significant correlation between verbal fluency and *H*_*corr*_ at the R-VWFA (r = 0.17, p > 0.60, Figure 3C), a right-hemispheric homolog of the VWFA that is not part of the language brain network (Cohen et al., 2003). These results suggest that, in older adults, verbal fluency is associated with neuronal selectivity in the VWFA, a key language region in the left ventral occipitotemporal cortex, but not the hippocampus and the R-VWFA. Similar results were obtained with Woodcock-Johnson III–Word Identification scores (see Figure 4), as well as using data from each of the three runs separately (data not shown here). In addition, the correlation coefficients between performance of two cognitive functions (episodic memory and verbal fluency) and *H*_*corr*_ at two brain regions (hippocampus and VWFA) were Z-transformed and shown in Figure 5, which revealed a clear interaction that is consistent with the double dissociation prediction.

Despite the fact that the VWFA was not found to be strongly activated in most previous neuroimaging studies of verbal fluency, here we provide evidence that letter verbal fluency is closely related to *H*_*corr*_ estimated neuronal selectivity in the VWFA, suggesting a critical role of the VWFA in letter verbal fluency. This is consistent with the lesion studies summarized above (Gleissner and Elger, 2001) as well as with the recent fMRI study that revealed a strong involvement of the VWFA during a letter verbal fluency test (Birn et al., 2010). In addition, the strong correlation between *H*_*corr*_ at the VWFA and verbal fluency suggests that neuronal selectivity (estimated via *H*_*corr*_) at the VWFA might be a reliable predictor of lexical access ability. This hypothesis is further supported by the significant correlation between *H*_*corr*_ at the VWFA and WJ Word ID scores (Figure 4). In contrast, the lack of significant correlation between verbal fluency and *H*_*corr*_ at the hippocampus suggests that, at least in healthy older adults, letter verbal fluency might not be a reliable measurement of neuronal function at the hippocampus (Baldo et al., 2006 but also see Gleissner and Elger, 2001). In addition, the lack of correlation between verbal fluency and *H*_*corr*_ at the R-VWFA is consistent with the argument that the R-VWFA is not critical for verbal fluency or language in general (Cohen et al., 2003), even though this R-VWFA region is often strongly activated by visually presented words and letters, with an even further increased activity in older adults (compared to young adults). These results thus argue against the neural dedifferentiation-related compensation hypothesis, but are in line with the theories of object recognition. These results provide further support that fMRI response amplitude, compared to neuronal selectivity (estimated via *H*_*corr*_ here), might be a poor measure of behavioral performance (Grill-Spector et al., 2006; Mahon et al., 2007). On the contrary, it has been proposed that neurons in the right fusiform area (similar to R-VWFA's location) might be involved in face processing and belong to a largely specialized functional region, the fusiform face area, or FFA (Kanwisher et al., 1997). Consistent with this, we and others found that face discrimination performance can be predicted by neuronal selectivity in the FFA (estimated via fMRI-A or *H*_*corr*_) (Jiang et al., 2006, 2013).

Taken together, the strong correlation between *H*_*corr*_ at the VWFA and letter verbal fluency performance suggests that VWFA is not only critical in reading (to process the visual inputs of letter strings), but also important for retrieving the letter string of words, in consistent with the hypothesis that VWFA might serve as a word “dictionary” responsible for the processing, learning, storing, and retrieving of word letter strings (Dehaene et al., 2005; Glezer et al., 2009, 2015), even in congenitally blind adults (Striem-Amit et al., 2012). However, it remains an open question about the role of VWFA in semantic or action verbal fluency, which does not necessarily involve the retrieval of word letter string.

## Discussion

Despite its significant scientific and clinical interest, relating individual differences in cognitive abilities to neural substrate in older adults remains a major challenge. Here by reanalyzing fMRI data from previously published work (Simon et al., 2011) in which subjects were participating in an implicit sequence learning task, we found that the novel *H*_*corr*_ technique (Jiang et al., 2013) can reliably estimate neuronal selectivity across different brain regions in healthy older adults with a single data set (even when subjects were performing an irrelevant task) and that individual differences in a specific cognitive function correlated with *H*_*corr*_ measures at the corresponding–but not other–brain regions. That is, we observed a double dissociation whereby individual differences in episodic memory performance were related to differences in neuronal selectivity in the hippocampus but not the VWFA, whereas the reverse was true for letter verbal fluency. In addition, there was no correlation between letter verbal fluency and *H*_*corr*_ measure at R-VWFA, the VWFA homologous region in the right hemisphere. These results suggest that individual differences in neuronal selectivity at specific brain regions might underlie individual differences in corresponding cognitive functions in healthy older adults. Furthermore, the ability to estimate neuronal selectivity across brain regions with a single data set and to estimate neuronal selectivity at a specific brain region (e.g., VWFA) even when subjects were performing an irrelevant task (implicit sequence learning) suggests that the novel *H*_*corr*_ technique has potential for studying healthy cognitive aging, and age-related neurological disease, such as Alzheimer's Disease, both associated with widespread change across brain regions.

Our findings here support theoretical work in cognitive aging suggesting that age-related neural dedifferentiation, i.e., a reduced neuronal selectivity in aged brain, is a key contributor of cognitive function in older adults (Li et al., 2001). In contrast, our results are not consistent with proposals that neural dedifferentiation might serve as a compensatory mechanism (Park and Reuter-Lorenz, 2009; Reuter-Lorenz and Park, 2010), but see (Reuter-Lorenz and Park, 2014). The neural dedifferentiation-related compensation hypotheses, such as the original STAC (the Scaffolding Theory of Aging and Cognition) model were inspired by findings from functional neuroimaging studies that often revealed greater activation of prefrontal and parietal regions in older adults (Gutchess et al., 2005; Davis et al., 2008), as well as an increase in bilateral recruitment (compared to more lateralized activity in younger adults) (Dolcos et al., 2002). Basically these theories argue that, to perform a specific cognitive task, age-related neural dedifferentiation might help older adults to recruit additional neurons/regions to compensate for the reduced neuronal function at the brain regions that are typically associated with the specific cognitive task/domain. While this compensatory mechanism might indeed underlie the increased activations in task circuits, such as frontal cortices in older adults (Reuter-Lorenz et al., 2000; Cabeza et al., 2002), the increased activity or recruitment of additional brain regions could also be due to factors like effort, attention, and task difficult levels, which are known to modulate fMRI response.

In addition, it is difficult to reconcile this dedifferentiation-related compensation hypothesis with findings from computational neuroscience of object recognition, and neuropsychological studies of patients with lesions.

First, computationally it is difficult to reconcile this compensation idea in the posterior regions that contain neurons highly selective to different features or object classes (e.g., face processing in FFA, vs. visual word processing in VWFA). That is, it is difficult to argue that FFA neurons selective to faces in young brain are now so broadly tuned in aged brain that these neurons can now be recruited to assist processing words or houses. Computationally the cost would be too high (i.e., the FFA neurons are now so broadly tuned that they are no longer highly efficient in processing faces) and the gain would be too small (i.e., again the FFA neurons are so broadly tuned that they would not be very effective for processing words or houses), suggesting that neural dedifferentiation at FFA is unlikely to be able to compensate for reduced word processing function due to neural dedifferentiation at VWFA, and vice versa, supporting by the lack of correlation between letter verbal fluency performance and *H*_*corr*_–measured neuronal selectivity in the right occipital-temporal region.

Second, lesions in the left hemisphere often lead to deficits in varying aspects of language processing, including aphasia. While patients might recruit homologous brain regions in the intact right hemisphere to retain some language capabilities (Basso et al., 1989; Turkeltaub et al., 2011), several functional neuroimaging studies of these left-hemisphere stroke patients have found that increased fMRI response at the right hemisphere homologous language regions may actually correlate with poorer language performance (Heiss et al., 1999; Postman-Caucheteux et al., 2010), suggesting the recruitment of homologous regions in the right hemisphere might be *detrimental* rather than *beneficial* in language processing, *even in patients with left hemisphere lesions along with an intact right hemisphere*. In addition, using the low frequency repetitive transcranial magnetic stimulation (rTMS) technique, which inhibits neural activity at targeted brain regions, studies have shown that inhibiting the right-hemisphere homologous language regions actually help with regaining language function in aphasia patients with left-hemisphere lesions (Hamilton et al., 2010), and can even help healthy adults to learn new words (Nicolo et al., 2016)–again against the dedifferentiation-related compensation hypothesis. Taken together, the data from these previous neuropsychology and rTMS studies suggest that, the increased bilateral fMRI response in healthy older adults (compared to younger adults) likely does not serve as a compensation mechanism, but rather it might be due to increased attentional load or task engagement, or decreased neuronal selectivity. While increased task engagement would lead to an increase in performance, decreased neuronal selectivity would correlate with a lower cognitive performance, along with a more complex relationship between increased attentional load and performance, which could be positive or negative.

Therefore here we argue that the present study provides evidence against some aspects of this compensation hypothesis, for two reasons: First, in contrast to the lack of correlation between performance and age-related neural dedifferentiation in previous studies, the double disassociation between episodic memory/verbal fluency and the hippocampus/VWFA in the present study suggest that cognitive function is directly, and positively, related to neuronal selectivity (measured via *H*_*corr*_) at corresponding, but not other brain regions, even in older adults; Second, the compensation hypothesis would predict a negative correlation between cognitive performance (e.g., verbal fluency) and neuronal selectivity at task-irrelevant regions (e.g., R-VWFA), but we did not observe any negative correlation (not even a trend) between varying cognitive abilities and *H*_*corr*_ at different brain regions. Therefore, we propose that age-related neural dedifferentiation has a *detrimental* rather than *compensatory* effect on cognitive performance in healthy older adults, i.e., the lower the neuronal selectivity at a brain region (e.g., hippocampus), the worse the corresponding cognitive function (e.g., episodic memory). The observed increase in activity or the recruitment of additional brain regions in healthy older adults might be more likely a consequence of cognitive decline, which would lead to neural dedifferentiation, increased attentional demand and task difficulty levels (all could lead to an increase in fMRI response), rather than a compensatory mechanism. However, compensation mechanisms might indeed help healthy older adults in some aspects of cognitive functions, such as the increased involvement of hippocampus in implicit learning in healthy older adults, which is typically associated with striatum in younger adults (Dennis and Cabeza, 2011). Future studies are needed to differentiate the increased activity due to age-related compensation or other factors (such as increased attentional demand), which might help to develop better cognitive training paradigms to preserve/improve cognitive function in aging.

Future studies are also needed to verify the Hcorr technique in a large and more heterogeneous sample, because the current sample is small. Longitudinal studies are also needed to examine the trajectory of change in neuronal selectivity from young to middle-aged to very older adults, along with detailed cognitive assessment, to provide definitive evidence on the relationship between neural dedifferentiation, aging, and cognitive performance. Furthermore, while the majority of previous studies on the correlation analysis of fMRI time series have been focusing on the correlations between time series at different brain regions—which have revealed impaired interregional connectivity due to aging (Chen et al., 2009), we and others have provided data suggesting that local voxel-wise correlations can help to uncover neuronal function/dysfunction at an intraregional level, using techniques like *H*_*corr*_ or ReHo (regional homogeneity) (Zang et al., 2004). For instance, using the fMRI ReHo and the [18F]-fluorodeoxyglucose (PET-FDG) techniques, a recent study revealed a tight correlation between the ReHo scores and the glucose metabolism across brain regions (Bernier et al., 2017), suggesting that local voxel-wise correlation might have the potential to serve as a non-invasive tool to study brain function/dysfunction. Therefore, combining interregional techniques like resting state functional connectivity analysis with this novel intraregional *H*_*corr*_ technique in future studies is expected to lead to a more complete and accurate understanding of the neural mechanisms underlying cognitive decline due to aging and/or age-related neurodegenerative disease.

Furthermore, compared to other well-established like techniques like fMRI-A and MVPA–both of which have been successfully applied to examine age-related decreases in neuronal selectivity (Carp et al., 2010; Goh et al., 2010; Lee et al., 2011), the novel *H*_*corr*_ technique offers several advantages for studying aging. First, both fMRI-A and MVPA techniques usually require lengthy scanning time, which is not ideal for research on older populations. In contrast, *H*_*corr*_ is highly sensitive and can be accomplished with much shorter scans (Jiang et al., 2013). Second, by design, previous techniques can only probe neural dedifferentiation in task-related brain regions, while aging is associated with changes across a broad range of brain regions (Levy, 1994; Hedden and Gabrieli, 2004; Grady, 2012). In contrast, *H*_*corr*_ has the potential to estimate neuronal selectivity across brain regions from a single data set, including resting state scans. More specifically, because previous single unit recording studies (Bair et al., 2001; Jermakowicz et al., 2009; Lin et al., 2014) found that pair-wise correlations between two neurons in the presence of visual stimuli can be detected in the absence of stimuli, thus the voxel-wise correlations and *H*_*corr*_ within a given region should be similar with or without a task. Similar neural mechanisms might also underlie the findings from fMRI studies of resting state (Fox and Raichle, 2007; Smith et al., 2009). These unique advantages suggest *H*_*corr*_ has strong potential for studying cognitive aging and cognitive neuroscience in general, and that this technique could be applied to rich sets of fMRI data that have already been collected. However, future studies that directly link neuronal activity and selectivity (through single or multiple unit recordings), *H*_*corr*_, and behavioral performance, are warranted to verify and validate this technique.

In summary, using a novel *H*_*corr*_ technique, we found a double dissociation in a sample of relatively homogeneous healthy older adults, such that episodic memory performance correlates with neuronal selectivity at hippocampus, but not VWFA, whereas verbal fluency shows the reverse pattern. In addition, there was no correlation between episodic memory and neuronal selectivity at the parahippocampal region, nor between verbal fluency and the R-VWFA. These results suggest that individual differences in cognitive abilities in healthy older adults are tightly linked to differences in neuronal selectivity at corresponding brain regions, and age-related neural dedifferentiation might be a *contributing* rather than *compensating* factor to age-related cognitive decline. However, this conclusion needs to be taken with caution as this study has several limitations. As this study was conducted from reanalyzing the data collected in a previously published study (Simon et al., 2011), the available behavioral and fMRI data was limited, the sample size was small, and the original experiment was not designed to test the hypotheses proposed here. Furthermore, the small sample size made it impossible to examine the potential difference due to gender, education, and other factors that might affect brain function and/or contribute to risks of certain age-related neurodegenerative diseases. Therefore, the present study should be treated as hypothesis generating rather than hypothesis testing, and future studies are warranted. However, the results we have reported here clearly demonstrate the sensitivity and applicability of this technique in studying cognitive aging, especially its heterogeneity, and we hope this study, as a proof or concept, will open a door for others to reanalyze the rich data that have already been collected, especially those with comprehensive neuropsychological assessments and a large and well-documented cohort.

## Ethics statement

Experimental procedures were approved by the Georgetown University's Institutional Review Board, and written informed consent was obtained from all subjects prior to the experiment.

## Author contributions

JP, DH, and JH designed the original study. JP conducted the original study. XJ reanalyzed the data. XJ, JP, DH, and JH wrote the paper.

### Conflict of interest statement

The authors declare that the research was conducted in the absence of any commercial or financial relationships that could be construed as a potential conflict of interest.
